# The optimum anticoagulation time after endovascular thrombectomy for atrial fibrillation-related large vessel occlusion stroke: a real-world study

**DOI:** 10.1007/s00415-022-11515-y

**Published:** 2023-01-03

**Authors:** Hongrui Ma, Ruiwen Che, Qihan Zhang, Wantong Yu, Longfei Wu, Wenbo Zhao, Ming Li, Di Wu, Chuanjie Wu, Xunming Ji

**Affiliations:** 1 Department of Neurology, Xuanwu Hospital, Capital Medical University, 45 Chang Chun St, Beijing, 100053 China; 2 China-America Institute of Neuroscience, Xuanwu Hospital, Capital Medical University, Beijing, 100053 China; 3 Department of Neurosurgery, Xuanwu Hospital, Capital Medical University, 45 Chang Chun St, Beijing, 100053 China

**Keywords:** Endovascular treatment, Acute ischemic stroke, Atrial fibrillation, Anticoagulant, sICH, ICH

## Abstract

**Objectives:**

To investigate the relationship between the initiation time of anticoagulation after endovascular treatment (EVT) and the outcomes in atrial fibrillation (AF)-related acute ischemic stroke (AIS) patients.

**Methods:**

In this prospective registry study, from March 2013 to June 2022, patients with anterior circulation territories AF-related AIS who underwent EVT within 24 h were included. The primary outcome was favorable [modified Rankin Scale (mRS) 0–1) at ninety days and the secondary outcome was hemorrhage events after anticoagulants. Factors affecting the outcomes were pooled into multivariate regression and ROC curve analysis.

**Results:**

Of 234 eligible patients, there were 63 (26.9%) patients achieved a favorable outcome. The symptomatic intracranial hemorrhage (sICH), ICH, and systemic hemorrhage events after anticoagulants occurred in 8 (3.4%), 28 (12.0%), and 39 (16.7%) patients, severally. A longer EVT to anticoagulation time (*p* = 0.033) was associated with an unfavorable outcome (mRS 3–6). An earlier EVT to anticoagulation time was the independent risk factor of sICH (*p* = 0.043), ICH (*p* = 0.005), and systemic hemorrhage (*p* = 0.005). There was no significant difference in recurrent AIS/ transient ischemic attack (TIA) or mortality among patients who started anticoagulation at ≤ 4 days, ≥ 15 days, or 4 to 15 days. The optimum cut-off for initiating anticoagulants to predict a favorable outcome and hemorrhage events was 4.5 days and 3.5 days after EVT, respectively.

**Conclusions:**

In AF-related AIS, the time of EVT to anticoagulation is an independent factor of the functional outcome and hemorrhage events after anticoagulation. The optimal initiate time of anticoagulant after EVT is 4.5 days.

**ClinicalTrialRegister:**

NCT03754738.

**Supplementary Information:**

The online version contains supplementary material available at 10.1007/s00415-022-11515-y.

## Introduction

As the independent risk factor of stroke, atrial fibrillation (AF) increased the hazard of stroke by 3–5 times [1, 2]. Anticoagulation was the effective access to the secondary prevention of AF-related strokes. It was deemed that two-thirds of AF-related strokes could be prevented by oral anticoagulants, which reduced the recurrence rate of ischemic stroke to 2.8% within 90 days [2–5]. Correspondingly, another risk was a 1.8% increase in intracranial hemorrhage (ICH) and symptomatic intracranial hemorrhage (sICH) due to reperfusion, impaired autoregulation, and disruption of the blood–brain barrier [1–4]. Indeed, there still existed a concern that early initiation increased the risk of hemorrhagic transformation, whereas delayed initiation left patients at risk for recurrent ischemic stroke [6]. The balance between the prevention of recurrent ischemic stroke and ICH particularly depends on the time and dose of anticoagulants [4].

Nevertheless, the administration of anticoagulants for stroke patients with AF was confusing and challenging. The American Heart Association-American Stroke Association (AHA-ASA) 2018 guidelines [7] recommended initiating oral anticoagulation within 4–14 days after AIS. The European Society of Cardiology 2016 [8] and European Heart Rhythm Association 2018 guidelines [9] recommend 1, 3, 6, and 12 days after the transient ischemic attack, minor, moderate, and severe strokes, respectively. While the efficacy of anticoagulants still depended on clinical practice in most cases due to the lack of data from clinical trials and few statements providing firm evidence. Particularly for acute ischemic stroke (AIS) patients with AF underwent EVT, due to the higher hemorrhage risk and severer clinical symptoms [10, 11], the initiation time of anticoagulants is still controversial so far. Here we conducted this study to gain better insight into the appropriate time for anticoagulation in AF-related stroke patients after endovascular thrombectomy (EVT).

## Methods

### Standard protocol approvals and informed consent

This study was based on a prospective randomized controlled trial registered on clinicaltrials.gov (NCT03754738). All enrolled AIS patients with AF receiving EVT had been approved by the Ethics Committee (NO. 2017030) of Xuanwu Hospital Capital Medical University, Beijing, China. The study was conducted according to the principles of the Declaration of Helsinki. All persons gave their informed consent prior to their inclusion in the study.

### Study population, inclusion, and exclusion criteria

In this prospective registry study, consecutive patients with AIS related to AF and treated with EVT from March 2013 to June 2022 in Xuanwu Hospital, Capital Medical University were enrolled. The inclusion criteria were: (1) diagnosis of AIS related to AF; (2) large vessel occlusion in the anterior circulation confirmed by computed tomographic angiography, magnetic resonance angiography, or digital subtraction angiography; (3) EVT initiated within 24 h after symptoms onset. (4) pre-stroke modified Rankin Scale (mRS) score ≤ 1 point. Patients who lost follow-up or without anticoagulation therapy after EVT were excluded.

### Interventions

The specific intervention strategies, the type of stent retrievers, and other devices were chosen at the discretion of the neuro-interventionists in our center who were professionally trained and certified in EVT. After EVT, patients were transferred to the neurological intensive care unit for further treatment. For patients with successful recanalization which was defined as the modified Thrombolysis in Cerebral Infarction (mTICI) perfusion score ≥ 2b, postoperative systolic blood pressure (SBP) was maintained between 100 and 140 mmHg to avoid hyper-perfusion as well as ICH. However, for those who did not achieve successful recanalization, the SBP was maintained between 140 and 180 mmHg to protect the collateral circulation. Other therapeutic regimens followed the recommendation of guidelines [12, 13]. The degree of neurological impairment of the patient was assessed by the National Institute of Health stroke scale (NIHSS). A higher NIHSS means a more severe disability [14]. Early cerebral ischemic change is assessed by Alberta Stroke Program Early CT Score (ASPECTS). A lower ASPECTS indicates more early ischemic changes detected on CT scans [15].

### Data collection

In our study, data were collected at baseline (clinical on admission) and follow-up visits (clinical or telephone) by a trained study physician. The collected data included demographics, medical history, HAS-BLED (hypertension, abnormal renal/liver function, stroke, bleeding history or predisposition, labile INR, elderly, drugs/alcohol concomitantly) score [14] (including 1 point for the index event in all patients), CHA2DS2-VASc (congestive heart failure, hypertension, age 75 years, diabetes mellitus, stroke/transient ischemic attack, vascular disease, age 65–74 years, sex category) score [16] (including 2 points for the index event in all patients), type of atrial fibrillation, clinical data included NIHSS, ASPECTS on admission and details of EVT, anticoagulation therapy and follow-up.

### Follow-up and outcomes

The overall follow-up period in this study was 90 days for each patient after the index event. Neurological function outcomes were assessed by modifying Rankins Scale (mRS) [17]. The mRS 0–1 was defined as a favorable outcome, and mRS 2–6 was considered to be an unfavorable outcome. The primary outcome of this study was considered to be favorable outcome. The secondary outcomes included new hemorrhage events (sICH, ICH, and systemic hemorrhage) secondary to anticoagulant therapy. The determination and grading standards of sICH and ICH followed the ECASSII criteria [18]. Outcomes were assessed by two independent investigators, and a third expert intervened when there was a disagreement. Patient status was followed up by telephone interviews or clinic visits when possible.

Patients enrolled in this study were divided into the favorable outcome group (mRS 0–1) and the unfavorable outcome group (mRS 2–6). Anticoagulants after EVT include low molecular weight heparin (LMWH) and/or direct oral anticoagulants (DOACs) including new oral anticoagulants (NOACs) (rivaroxaban, dabigatran etexilate, etc.) or Vitamin K antagonists (VKAs) (warfarin, etc.).

### Statistical analyses

The Shapiro–Wilk test was used for testing the normality of continuous variables. Descriptive statistics were presented as mean (standard deviation [SD]) for normally distributed continuous variables or medians (interquartile range [IQR]) for non-normally distributed continuous variables and as percentages for categorical variables. The Student’s *t* test was conducted for two groups of normally distributed continuous variables or the Mann–Whitney *U* test for continuous non-normally distributed continuous variables. The Chi-square test or Fisher's exact test was conducted for categorical variables. Furthermore, univariate and binary logistic regression analysis was performed to explore the risk factors associated with the outcomes. The ROC curve was drawn to test the sensitivity, specificity, and optimal cutoff to predict the outcomes. The significance level was set at *p* < 0.05 (two-sided). Statistical analyses were performed with SPSS 26.0 (IBM Corp).

## Results

### Demographic characteristics

**Fig. 1:**
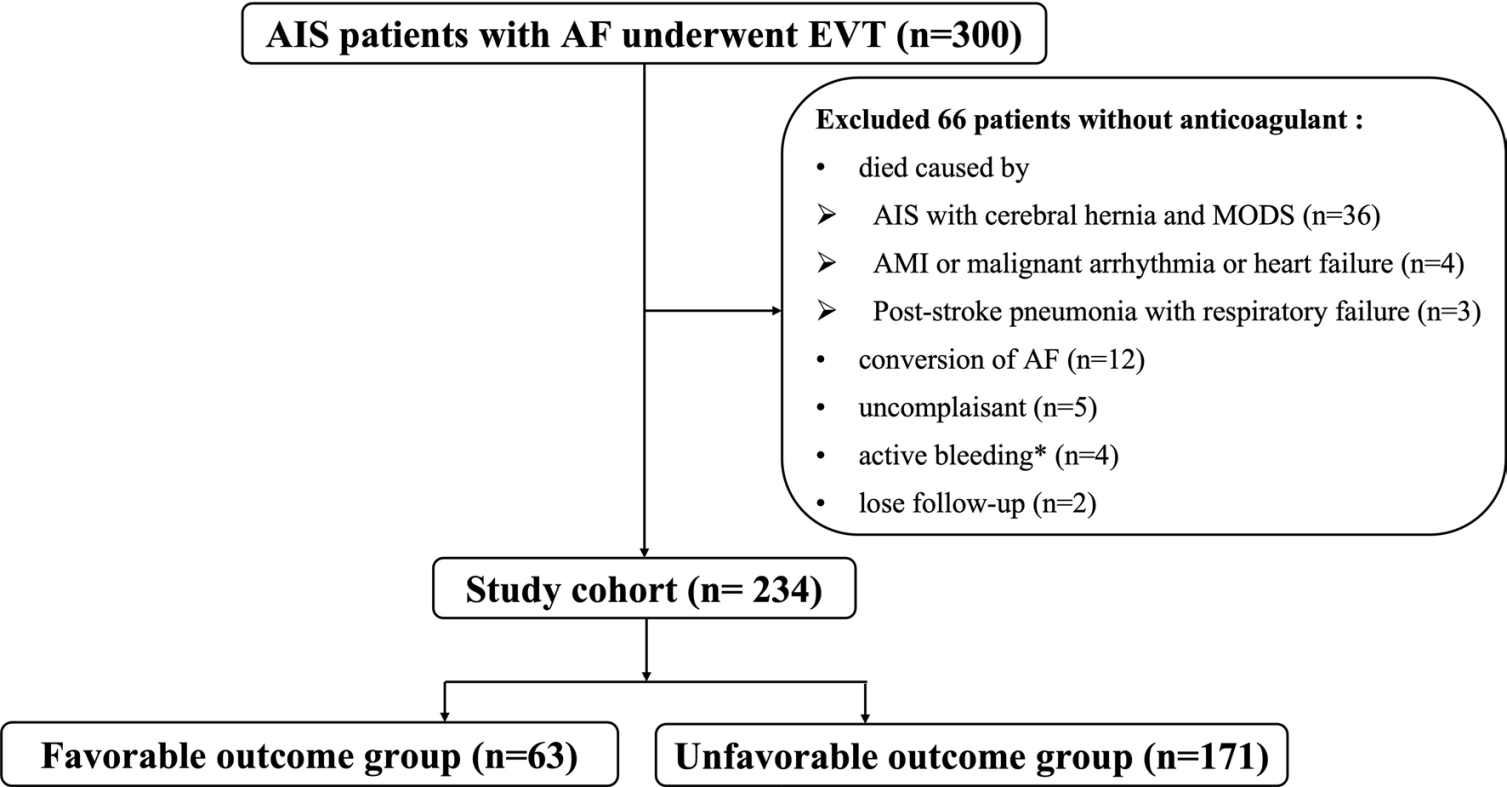
Flow diagram. The study enrolled 300 individuals with anterior circulation territories AF-related AIS who underwent EVT within 24 h. A total of 66 patients without anticoagulants after EVT were excluded. The final study cohort included 234 eligible patients, and there were 63 patients divided into the favorable outcome group and 171 patients into the unfavorable outcome group according to the mRS at ninety-day after EVT. *AF* atrial fibrillation, *AIS* acute ischemic stroke, *EVT* endovascular thrombectomy, *MODS* multiple organ dysfunction syndromes, *AMI* acute myocardial infarction. *Continuous active bleeding due to severe stress ulcer of the digestive tract; Favorable outcome: mRS 0–1; Unfavorable outcome: mRS 3–6

A total of 300 AIS patients with AF underwent EVT therapy from March 2013 to June 2020 and were consecutively enrolled in this study. We excluded 66 patients without any anticoagulation therapy including 43 patients who died due to AIS with cerebral hernia and multiple organ dysfunction syndromes (MODS), acute myocardial infarction (AMI), malignant arrhythmia, heart failure, and post-stroke pneumonia with respiratory failure; twelve patients with the conversion of AF, five patients who did not follow the medical advice, four patients who had severe stress ulcer of the digestive tract, and two patients losing follow-up. Finally, the clinical data of 234 eligible patients were pooled into the final analysis. We divided all of the patients into the favorable outcome group (mRS 0–1) and the unfavorable outcome group (mRS 3–6) (Fig. 1). There were 63 (26.9%) achieved a favorable outcome. The baseline characteristics data between the two groups were presented in Table 1. A total of 30 patients who were included in this study died at 90-day follow-up and the causes of death are shown in Supplemental Fig. 1.

**Table 1:** Baseline characteristics of favorable outcome group and unfavorable outcome group

	All patients (*n* = 234)	Favorable outcome group (*n* = 63)	Unfavorable outcome group (*n* = 171)	*p* value
Age, mean (SD), y	71 (10.15)	68 (10.19)	72 (9.99)	0.017*
Female, *n* (%)	102 (43.6)	33 (52.4)	69 (40.4)	0.100
BMI, mean (SD), kg/m^2^	24.90 (3.63)	25.00 (3.50)	24.86 (3.69)	0.795
Medical history, *n* (%)				
Hypertension	149 (63.7)	31 (49.2)	118 (69)	0.005**
Diabetes mellitus	57 (24.4)	7 (11.1)	50 (29.2)	0.004**
Coronary heart disease	63 (26.9)	6 (9.5)	57 (33.3)	< 0.001***
Ischemic stroke	59 (25.2)	10 (15.9)	49 (28.7)	0.046*
Smoke	58 (24.8)	13 (20.6)	45 (26.3)	0.372
Kidney failure	21 (9)	2 (3.2)	19 (11.1)	0.060
AF details				
Previous AF, *n* (%)	174 (74.4)	51 (81)	123 (71.9)	0.161
Previous anticoagulation, *n* (%)	49 (20.9)	23 (36.5)	26 (15.2)	< 0.001***
Nonvalvular atrial fibrillation, *n* (%)	203 (86.8)	50 (79.4)	153 (89.5)	0.043**
HAS-BLED, mean (SD), points	2.33 (1.17)	1.95 (1.11)	2.47 (1.17)	0.002**
CHA_2_DS_2_-VASc, mean (SD), points	3.64 (1.66)	3.21 (1.47)	3.80 (1.70)	0.015*
AF types				
Paroxysmal AF, *n* (%)	60 (25.6)	16 (25.4)	44 (25.7)	0.371
Persistent AF, *n* (%)	151 (64.5)	38 (60.3)	113 (66.1)	
Permanent AF, *n* (%)	23 (9.8)	9 (14.3)	14 (8.2)	
Clinical data on admission				
NIHSS, median (P25, P75), points	15 (12, 18)	13 (10, 18)	16 (13, 19)	< 0.001***
ASPECTS, median (P25, P75), points	9 (8, 10)	9 (8, 10)	9 (8, 10)	0.053
SBP, mean (SD), mmHg	145 (25.03)	138 (24.58)	148 (24.77)	0.010*
Glucose, mean (SD), mmol/L	8.09 (3.37)	6.59 (2.07)	8.64 (3.59)	< 0.001***
INR, median (IQR)	1.12 (0.21)	1.12 (0.25)	1.11 (0.19)	0.728
Creatinine, median (SD), umol/L	70 (30.12)	64 (16.17)	72 (33.63)	0.070
Urea, median (SD), mmol/L	6.13 (2.40)	5.51 (1.85)	6.36 (2.54)	0.016*
Procedures of IVT and EVT				
Intravenous thrombolysis, * n* (%)	79 (33.8)	18 (28.6)	61 (35.7)	0.308
ICA occlusion, * n* (%)	85 (36.3)	14 (22.2)	71 (41.5)	0.006**
General anesthesia, * n* (%)	52 (22.2)	8 (12.7)	44 (25.7)	0.033*
Intra-arterial thrombolysis, * n* (%)	4 (1.7)	1 (1.6)	3 (1.8)	1.000
NOP, mean (SD), times	1.15 (1.03)	0.89 (0.85)	1.24 (1.07)	0.020**
Thrombus aspiration, * n* (%)	182 (77.8)	50 (79.4)	132 (77.2)	0.723
Stent implantation, * n* (%)	13 (5.6)	3 (4.8)	10 (5.8)	1.000
Balloon dilatation, * n* (%)	16 (6.8)	1 (1.6)	15 (8.8)	0.101
OPT, median (P25, P75), min	334 (234.25, 465)	325 (196, 410)	421 (313, 540)	0.122
ORT, median (P25, P75), min	401 (306.25, 515.25)	380 (275, 465)	421 (313, 540)	0.016*
Residual stenosis, * n* (%)	24 (10.3)	4 (6.3)	20 (11.7)	0.232
mTICI ≥ 2b, * n* (%)	207 (88.5)	59 (93.7)	148 (86.5)	0.132
Reocclusion with 72 h, * n* (%)	19 (8.1)	0 (0)	19 (11.1)	0.006**
Decompressive craniectomy, * n* (%)	11 (4.7)	0 (0)	11 (6.4)	0.087
sICH after EVT	25 (10.3)	0 (0)	25 (14.6)	0.001**
Any ICH after EVT	109 (44.9)	20 (31.7)	99 (57.9)	< 0.001***
Anticoagulation after EVT				
EVT to anticoagulation time, mean (SD), day	7.40 (7.59)	5.10 (4.26)	8.25 (8.35)	0.005**
sICH after anticoagulation	8 (3.4)	0 (0)	8 (4.7)	0.180
Any ICH after anticoagulation	28 (12.0)	3 (4.8)	25 (14.6)	0.039*
Systemic hemorrhage after anticoagulation	39 (16.7)	7 (11.1)	32 (18.7)	0.166

*BMI* body mass index, *AF* atrial fibrillation, *NIHSS* National Institute of Health stroke scale, *ASPECTS* Alberta Stroke Program Early CT Score, *SBP* systolic blood pressure, *INR* international normalized ratio, *EVT* endovascular treatment, *ICA* internal carotid artery, *NOP* number of passes, *OPT* onset to puncture time, *ORT* onset to reperfusion time, *mTICI* modified Thrombolysis in Cerebral Infarction Score, *sICH* symptomatic intracranial hemorrhage, *VET* venous thrombosis

^***^p < 0.001; **p < 0.01; *p < 0.05

The average age of the favorable outcome group (68 ± 10.19) was younger than those of the unfavorable outcome group (72 ± 9.99) (*p* = 0.017). The gender and BMI between the two groups were well balanced. Patients in the favorable outcome group had less ratios of hypertension (49.2% vs 69%, *p* = 0.005), diabetes mellitus (11.1% vs 29.2%, *p* = 0.0.04), coronary heart disease (9.5% vs 33.3%, *p* < 0.001) and ischemic stroke (15.9% vs 28.7%, *p* = 0.046). No significant difference in smoke and kidney failure ratio between the two groups. As for atrial fibrillation (AF), a percentage of 74.4% of patients in this study had a history of AF, and the other patients were first detected with AF upon admission to the AIS event. A major proportion (86.8%) of patients suffered from nonvalvular AF. Patients in the favorable outcome group had a larger percentage of anticoagulants history before the AIS event (36.5% vs 15.2, *p* < 0.001), and the less score of HAS-BLED (1.95 ± 1.11 vs 2,95 ± 1.17, *p* = 0.002) and CHA2DS2-VASc (3.21 ± 1.47 vs 3.80 ± 1.70, *p* = 0.015) than those of unfavorable outcome group. Patients in the favorable group had a lower proportion of nonvalvular atrial fibrillation (79.4% vs 89.5%, *p* = 0.043). Over half (64.5%) of patients had persistent AF. No significant difference between the two groups in the types of AF.

The clinical data about the details for AIS on baseline are also listed in Table 1. Patients in the favorable outcome group had lower levels of NIHSS, systolic blood pressure, random serum glucose, and serum urea than in the unfavorable outcome group. The levels of ASPECTS, international normalized ratio (INR), and creatinine were well-balanced between the two groups.

There was about a third of (33.8%) patients in this study received bridging treatment (IVT followed by EVT). There was no statistical difference between the two groups in receiving bridging treatment or direct EVT. For patients in the favorable outcome group, less percentage of internal carotid artery occlusion (22.2% vs 41.5%, *p* = 0.006) and general anesthesia (12.7% vs 25.7%, *p* = 0.033), less numbers of passes (NOP) (0.89 ± 0.85 vs 1.24 ± 1.07, *p* = 0.020), less duration of onset to reperfusion time (median: 380 vs 421, *p* = 0.016), less reocclusion ratio within 72 h (0 vs 11.1%, *p* = 0.006) were indicated than those of the unfavorable outcome group. No pronounced differences were found in the perspectives of adjuncts to endovascular therapy (intra-arterial thrombolysis, thrombus aspiration, stent implantation, and balloon dilatation) and onset to puncture time (OPT) between the two groups. For the reperfusion status after EVT, a small part of patients received residual stenosis and most patients (88.5%) patients achieved good reperfusion (mTICI ≥ 2b).

The safety outcomes of anticoagulation therapy after EVT are shown in Table 1. The average time of initiating anticoagulants after EVT in this cohort of patients was 7.4 days, and patients who got a favorable outcome had earlier anticoagulant time than the unfavorable outcome group (5.1 ± 4.26 vs 8.25 ± 8.35, *p* = 0.005). The incidence of sICH and any ICH events after EVT were 10.3% and 44.9% among all the recruited patients. The ratio of sICH (0 vs 14.6%, *p* = 0.001) and any ICH (31.7% vs 57.9, *p* < 0.001) in the favorable outcome group were significantly lower than those of the unfavorable outcome group. After EVT, the newborn events of sICH, any ICH, and systemic hemorrhage after anticoagulation occurred in 3.4%, 12%, and 16.7% of patients, respectively. Patients in the favorable outcome group got less possibility of any ICH event after anticoagulation (4.8% vs 14.6%, *p* = 0.039) than those in the unfavorable outcome group. No differences were found between the two groups on sICH and systemic hemorrhage after anticoagulation.

The confounding factors including factors found in univariate analysis (*p* value < 0.05) and risk factors that might be associated with the functional outcomes were pooled into the binary logistic regression to find the independent factors that affect the favorable/unfavorable outcome. As shown the data in Table 2 and the corresponding forest plot, coronary heart disease (adjusted OR: 0.214; 95% CI 0.068–0.670, *p* = 0.008), NIHSS on admission (adjusted OR: 0.916; 95% CI 0.844–0.995, *p* = 0.038), random serum glucose (adjusted OR: 0.794; 95% CI 0.674–0.974, *p* = 0.027), ICA occlusion (adjusted OR: 0.417; 95% CI 0.177–0.980, *p* = 0.045), number of passes (adjusted OR: 0.555; 95% CI 0.342–0.900, *p* = 0.017), onset to reperfusion time (adjusted OR: 0.997; 95% CI 0.995–1.000, *p* = 0.028) and EVT to anticoagulation time (adjusted OR: 0.923; 95% 0.858–0.994) were all the independent risk factors of unfavorable outcome.

**Table 2:**
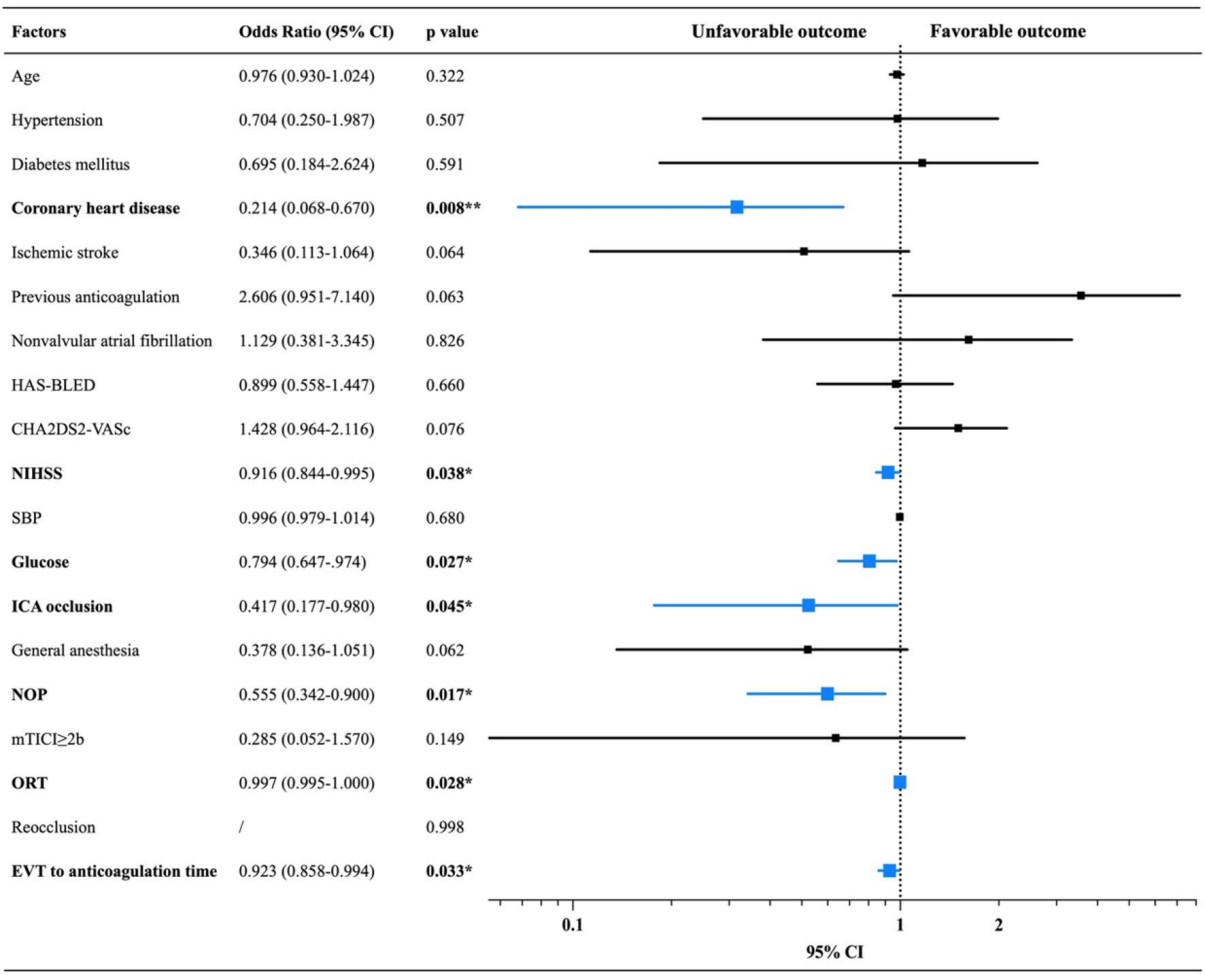
Binary logistic regression analysis of factors associated with favorable/unfavorable outcome

*NIHSS* National Institute of Health stroke scale, *ICA* internal carotid artery, *NOP* number of passes, *mTICI* modified Thrombolysis in Cerebral Infarction Score, *ORT* onset to reperfusion time, *EVT* endovascular treatment

^***^p < 0.001; **p < 0.01; *p < 0.05

To investigate the risk factors of hemorrhage events after EVT followed by anticoagulation, age, NIHSS on admission, INR on admission, ICA occlusion, number of passes, onset to reperfusion time, previous anticoagulation history, and EVT to anticoagulation time were pooled into the binary logistic regression analysis. As presented in data of Table 3 and the forest plots, EVT to anticoagulation time was the independent risk factor of sICH (adjusted OR: 0.454; 95% CI 0.219–0.940, *p* = 0.034), ICH (adjusted OR: 0.778; 95% CI 0.654–0.926, *p* = 0.005) and systemic hemorrhage (adjusted OR: 0.889; 95% CI 0.818–0.965, *p* = 0.005) after anticoagulation therapy.

**Table 3:**
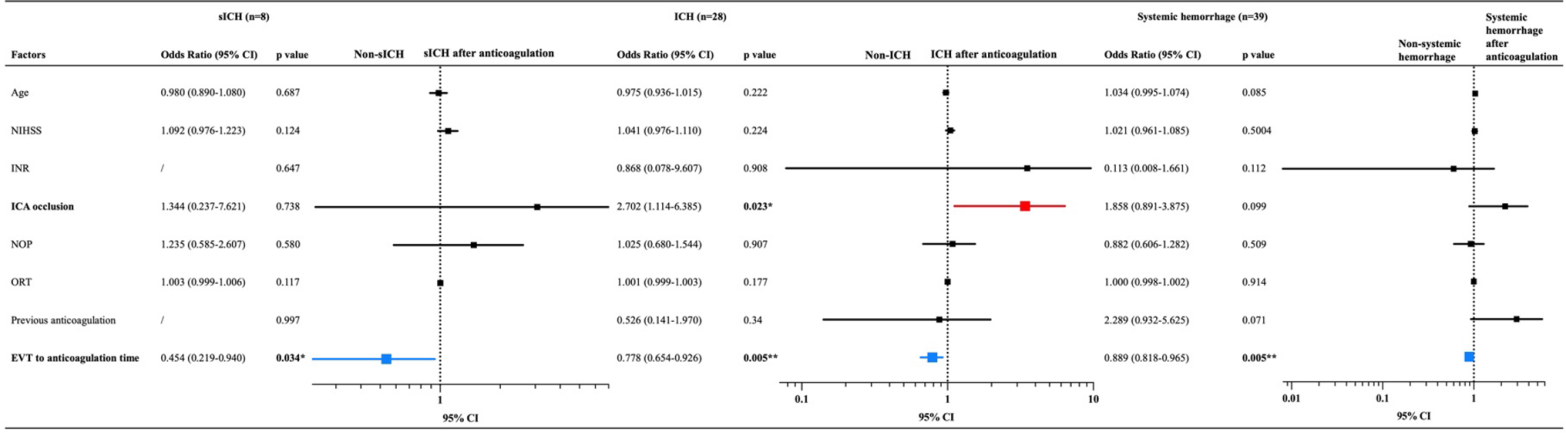
Binary logistic regression analysis of factors associated with hemorrhage events after anticoagulation

*sICH* symptomatic cerebral hemorrhage, *NIHSS* National Institute of Health stroke scale, *INR* international normalized ratio, *ICA* internal carotid artery, *NOP* number of passes, *ORT* onset to reperfusion time, *EVT* endovascular treatment

^***^p < 0.001; **p < 0.01; *p < 0.05

**Fig. 2:**
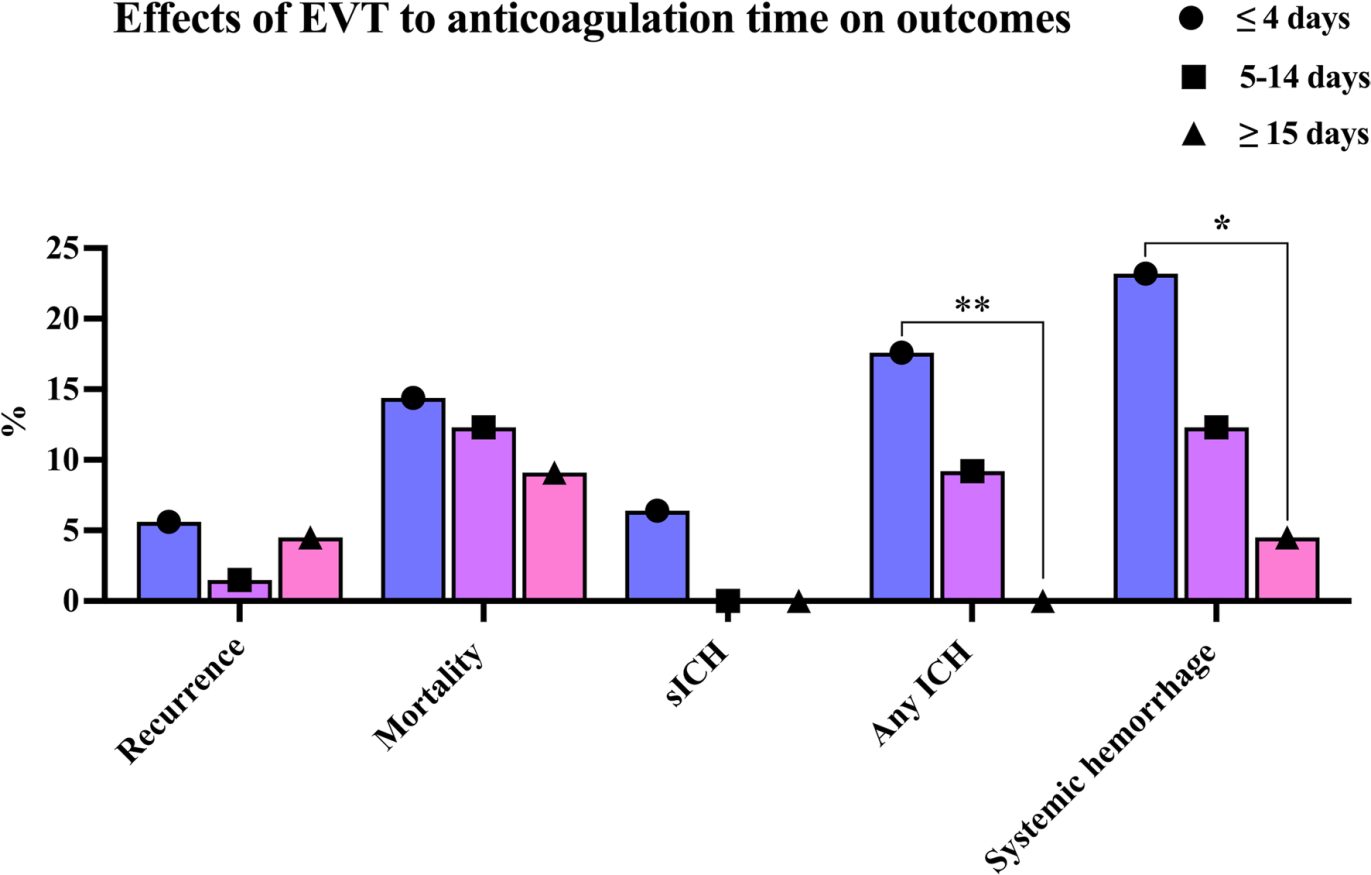
Incidence of efficacy and safety outcomes at different times. There was no significant statistical difference in recurrent AIS/TIA, mortality, and sICH events at ninety-day follow-up among the three groups. The incidences of any ICH and systemic hemorrhage of patients who received early anticoagulation (EVT to anticoagulation ≤ 4 days) were higher than those who received late anticoagulation (EVT to anticoagulation ≥ 15 days). *EVT* endovascular thrombectomy, *AIS* acute ischemic stroke, *TIA* transient ischemic attack, *sICH* symptomatic intracranial hemorrhage, *ICH* intracranial hemorrhage. **p < 0.01; *p < 0.05

To further investigate the effects of EVT on anticoagulation time on the efficacy outcome (AIS/TIA recurrence) and the safety outcomes (mortality, sICH, ICH, and systemic hemorrhage) at the 90-day follow-up, all 234 patients were divided into three groups according to the duration between EVT and anticoagulants initiation. As shown in Table 4, there were 125 patients with EVT to anticoagulation time ≤ 4 days identified in the early anticoagulation group. Some 66 patients with EVT to anticoagulation time from 5 to 14 days was a group of appropriate time zone according to the guidelines [7]. The other 43 patients with EVT to anticoagulation time ≥ 15 days were considered to be divided into the late anticoagulation group. The AIS/TIA recurrence ratio of the three groups identified above were 5.6%, 1.5%, and 4.5%, respectively. The mortality of the three groups was 14.4%, 12.3%, and 9.1%, respectively. One-way ANOVA analysis indicated that there was no significant difference among the three groups in AIS/TIA recurrence ratio, mortality, and sICH. Patients who received anticoagulation ≤ 4 days after EVT had a significantly higher incidence of any ICH and systemic hemorrhage events than those who initiated anticoagulants ≥ 15 days (Table 4 and Fig. 2).

**Table 4:** Effects of EVT to anticoagulation time on recurrence and mortality

Outcomes	EVT to anticoagulation time			*p* value
	≤ 4 days (*n* = 125)	5–14 days (*n* = 66)	≥ 15 days (*n* = 43)	
Recurrence of AIS or TIA (%)	5.6	1.5	4.5	0.357
Mortality (%)	14.4	12.3	9.1	0.656
sICH (%)	6.4	0	0	0.085
Any ICH (%)	17.6	9.2	0	**0.006**
Systemic hemorrhage (%)	23.2	12.3	4.5	**0.01**

**Fig. 3:**
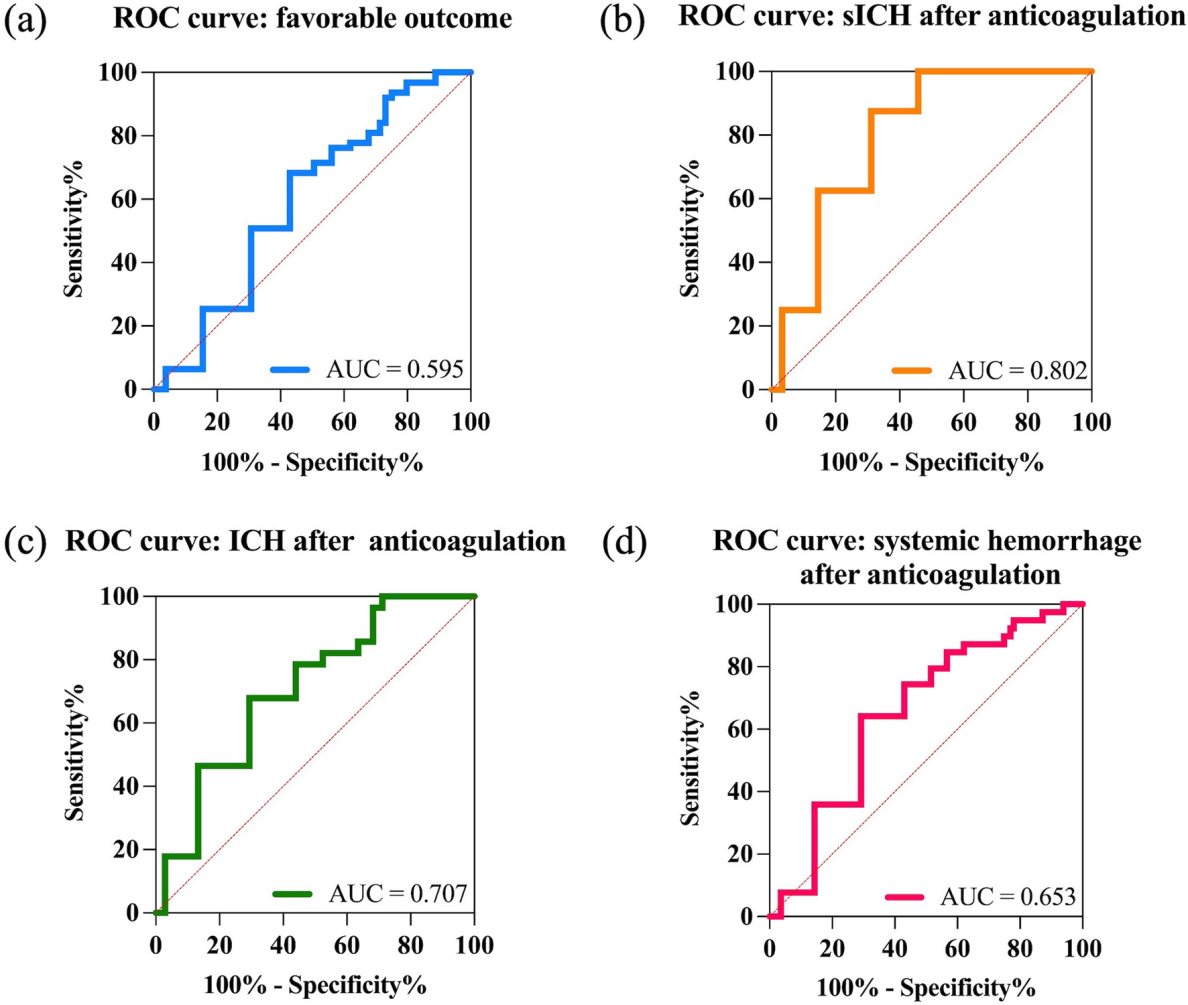
The ROC curves for the efficacy and safety outcomes. The AUC values of EVT to anticoagulation time to predict favorable outcome (**a**), sICH (**b**), ICH (**c**), and systemic hemorrhage (**d**) were 0.595, 0.802, 0.707, and 0.653, respectively. *AUC* area under the curve, *sICH* symptomatic intracranial hemorrhage, *ICH* intracranial hemorrhage. Central Illustration Anticoagulation time after endovascular thrombectomy for atrial fibrillation-related acute ischemic stroke

The ROC curves and AUC (area under the curve) of EVT anticoagulation time for predicting the favorable outcome and each of the safety outcomes (sICH, ICH, and systemic hemorrhage after anticoagulation) were shown in Fig. 3. From the parameters of the ROC curve indicated in Table 5, the cut-off values (Youden index) of EVT to anticoagulation time for predicting the favorable outcome, sICH, ICH, and systemic hemorrhage were 4.5 days [68.25% of sensitivity, 52.05% of specificity, 0.344 of positive predictive value (PPV) and 0.817 of negative predictive value (NPV)], 4.5 days (100% of sensitivity, 48.23% of specificity, 0.064 of PPV and 1.000 of NPV), 3.5 days (67.86% of sensitivity, 62.14% of specificity, 0.196 of PPV and 0.934 of NPV) and 3.5 days (64.1% of sensitivity, 63.08% specificity, 0.260 of PPV and 0.898 of NPV).

**Table 5:** Parameters of ROCs for the efficacy and safety outcomes

Outcomes	Std.Error	95% CI	*p* value	Sensitivity (%)	Specificity (%)	PPV	NPV	Cut-off value (day)
Favorable outcome	0.039	0.519–0.671	0.026*	68.25	52.05	0.344	0.817	4.5
sICH after anticoagulation	0.055	0.694–0.909	0.004**	100	48.23	0.064	1.000	3.5
ICH after anticoagulation	0.047	0.614–0.800	< 0.001***	67.86	62.14	0.196	0.934	3.5
Systemic hemorrhage after anticoagulation	0.044	0.566–0.740	0.003**	64.1	63.08	0.260	0.898	3.5

*sICH* symptomatic intracranial hemorrhage, *ICH* intracranial hemorrhage, *PPV* positive predictive value, *NPV* Negative predictive value

^***^p < 0.001; **p < 0.01; *p < 0.05

## Discussion

**Fig. 4:**
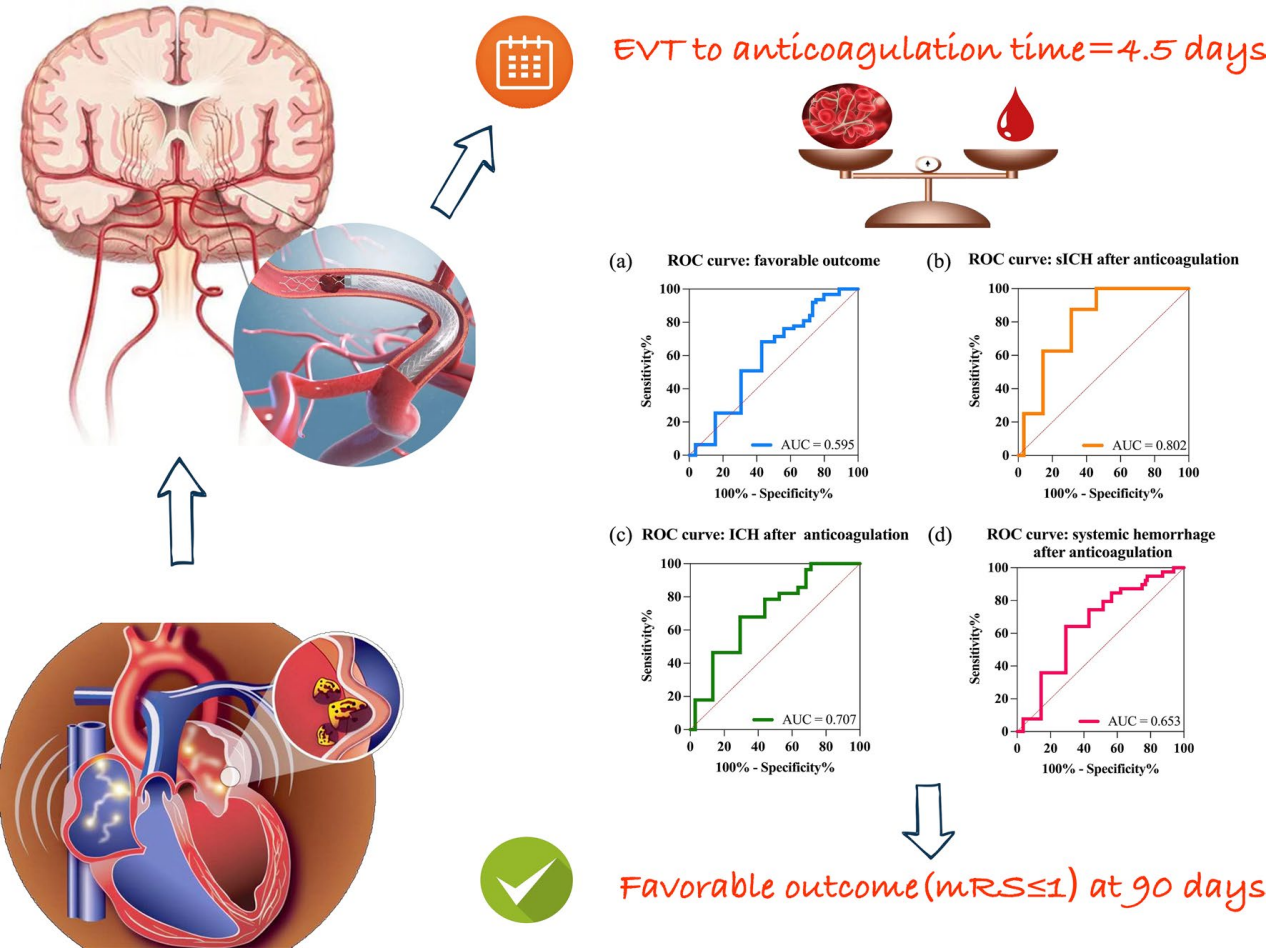
Central Illustration. For acute ischemic stroke patients associated with atrial fibrillation who received endovascular thrombectomy (EVT), the initiation time of anticoagulants after EVT is an independent factor that affects the incidence of the favorable outcome (mRS 0–1) and hemorrhage events at ninety-day follow-up. Early anticoagulation (EVT to anticoagulation ≤ 4 days) increases the risk of intracranial hemorrhage and systemic hemorrhage events more than late anticoagulation (EVT to anticoagulation ≥ 15 days). The optimum time for initiating anticoagulants after EVT to achieve a favorable outcome is 4.5 days

The population in this study included AF-related patients with large vessel occlusion who underwent EVT. The incidence of sICH and any ICH events after EVT of patients in this cohort study were 10.3% and 44.9%, which was slightly higher than the average incidences of the five RCTs. The average NIHSS on admission was 15 (IQR12-18). Furthermore, a part of the patients received bridge therapy, which might be sicker patients with large strokes, and with a high risk of hemorrhagic complications. It is possible that more factors such as IVT, drug administration during thrombectomy, antithrombotic therapy, endovascular damage after thrombectomy, ischemia–reperfusion injury, etc. would affect ICH and other complications exist. Therefore, it undoubtedly increased the complexity and difficulty of anticoagulation therapy for AF-associated AIS followed by EVT. In this case, this observational study firstly identified that for AF-related AIS patients who received EVT, a history of coronary heart disease, higher NIHSS on admission, higher serum glucose level on admission, ICA occlusion, a larger number of passes, and a longer onset to reperfusion time were associated with the unfavorable outcome. These results were consistent with the previous research. Moreover, a shred of new-found evidence in this study was that a longer EVT to anticoagulation time was the independent risk factor that was associated with the efficacy and safety outcomes at 90-day follow-up after AIS onset (Fig. 4).

Based on the guidelines and the clinical practice in large clinical studies [19]. We further divided the eligible patients into the early anticoagulation group, appropriate anticoagulation group, and late anticoagulation group, according to the time point of anticoagulation initiation followed by EVT. The result in this part suggested that the rate of hemorrhage events (any ICH and systemic hemorrhage) in the early anticoagulation group was significantly higher than that in the late anticoagulation group. Studies have shown that, from the pathological perspective, there exists damage to neurovascular units, in other words, named blood–brain barrier dysfunction (BBBD) in the early stage of stroke patients, especially in the situation of ischemia–reperfusion after thrombectomy, such damage may be aggravated. Therefore, early anticoagulation may increase the ICH burden caused by BBBD [1, 4]. Our study was consistent with previous studies suggesting that early anticoagulation increases the risk of ICH more than late anticoagulation.

However, there wasn’t a piece of potent evidence to demonstrate that a late anticoagulation strategy benefited the decrease in recurrent ischemic cerebrovascular disease, mortality, and sICH event in our study (*p* > 0.05) (Fig. 2). It might be limited by the smaller sample size, it neither obvious to observe the incidences in such a short follow-up time. While in this case, we still got a trend that the incidences of the safety outcomes in Fig. 2 were negatively correlated with the prolongation of the EVT to anticoagulation time. Especially, during 5–14 days of EVT to anticoagulation time, the recurrence rate of ischemic cerebrovascular disease is the lowest. Hence, it’s quite necessary to explore a balanced time point or period for achieving a favorable outcome and avoiding adverse events as well.

Furthermore, according to the result of our studied longitudinal cohort, we found that the optimum initiation time of anticoagulants after EVT for prediction of the favorable outcome (mRS 0–1) was 4.5 days, and 3.5 days for the hemorrhage events (sICH, any ICH, systemic hemorrhage). These two cut-off times also demonstrated that firstly, initiation of anticoagulant within four days after EVT does not improve the functional outcome of patients. This was consistent with the conclusions of previous studies that among patients with AF and LVO stroke necessitating EVT, adequacy of anticoagulation does not significantly affect the chances for a favorable outcome [20]. Correspondingly, it increases the risk of hemorrhage. Thus, we concluded that although over half (53.4%) of patients received early anticoagulation therapy (Table 4), the safety and efficacy of early anticoagulation still needed further verification.

Indeed, the debates on the optimal timing of anticoagulants after stroke existed for a long time and a series of studies focused on this issue. For example, multicenter data of Maurizio et.al [21] showed that the best time for initiating anticoagulation treatment for secondary stroke prevention is 4 to 14 days from stroke onset. While, recently, a multicenter study from Shadi et al. [22] suggested that anticoagulation 4–14 days after onset was not associated with reduced ischemic and hemorrhagic outcomes for cardioembolic stroke patients. A study focused on mild-to-moderate AF-associated ischemic stroke showed a low risk of surrogate ICH and sICH about early DOACs-initiation (median 3–5 days) [23]. Moreover, previous studies showed that among AF-related AIS patients receiving anticoagulation, and early anticoagulation of DOACs (≤ 7 days), the risk of ICH appears to be low [19]. The reason for the differences is that complicated factors account for an anticoagulant time such as infarct size, hemorrhagic transformation, or high-risk features on echocardiography need to be considered, which would delay the clinical decision to start anticoagulation.

In this study, there were 86.8% of patients suffered from NVAF. The DOACs or VKAs can both be used as the first-line therapy for the secondary prevention of ischemic stroke in nonvalvular atrial fibrillation (NVAF) patients [24]. However, the VKAs and DOACs have important differences in pharmacokinetic and pharmacodynamic effects. For example, the peak antithrombotic effect of DOACs occurs within a few hours after a single dose while the onset action of VKA is delayed to a few days [25]. Studies had shown that DOACs had lower risks of the composite endpoint of ischemic and hemorrhagic outcomes as compared to VKAs [26] and it is comparable or superior to VKAs for atrial fibrillation and venous thromboembolism [27–31]. Thus, a future perspective on the choice of DOACs or VKAs should be considered to achieve better outcomes.

## Study strengths and limitations

The strengths of our study include the clinical practice based on a real-world study. It provides relatively objective and truthful evidence of an anticoagulation strategy focusing on AF-related AIS patients who underwent EVT.

Our study had several limitations. Firstly, since this study was not a randomized controlled trial, the results were possibly influenced by some confounders. Although patients were continuously enrolled, there existed selection bias to therapy causes in actual clinical practice. For example, in clinical practice, clinicians tended to select patients with a low risk of ICH for early anticoagulation and delay the initiation time of anticoagulants according to the large volume of cerebral infarction and severe degree of hemorrhage transformation and such selection bias is indeed inevitable. Meanwhile, as reported by Arboix’s study [32], the model to predict the outcomes (e.g. in-hospital mortality) in cardioembolic cerebral infarction patients included several important clinical features such as altered consciousness, limb weakness, and history of congestive heart failure. Thus, to control the influence of confounding factors on the results of our study as much as possible, univariate regression analysis and logistic regression analysis were used for bias correction. Under this circumstance, it might be a new clue that early anticoagulation was related to the increase in the risk of ICH, and the data represented real-world data is valuable. However, it could be more meaningful to appeal to the further data from a randomized control study that really matters.

Secondly, given the relatively small sample size and the low event rate such as recurrent ischemic stroke or TIA, our study may have been underpowered and it’s more difficult to expand subgroup analysis. All in all, randomized controlled trials assessing the optimal time and types of anticoagulants after EVT for AIS patients with AF are warranted to guide clinical practice in the future.

## Conclusions

In conclusion, EVT to anticoagulation time is an independent factor that affects the functional outcome and hemorrhage events after the anticoagulation strategy in AIS patients with atrial fibrillation who received EVT. Early anticoagulation increases the risk of ICH more than late anticoagulation. The optimum initiate time of anticoagulant for achieving a favorable outcome is 4.5 days after EVT in AF-associated AIS patients.

## Supplementary Information

Below is the link to the electronic supplementary material.

Supplementary file1 (TIFF 178 KB)

Supplementary file2 (DOCX 13 KB)

## Data Availability

The data that support the findings of this study are available on request from the corresponding author, [C.J.W and X.M.J]， upon reasonable request.
