# Supplementary figures and images for: The optimum anticoagulation time after endovascular thrombectomy for atrial fibrillation-related large vessel occlusion stroke: a real-world study
Source: J Neurol. 2023 Jan 3;270(4):2084–95. doi: 10.1007/s00415-022-11515-y (PMC10025205; doi:10.1007/s00415-022-11515-y)

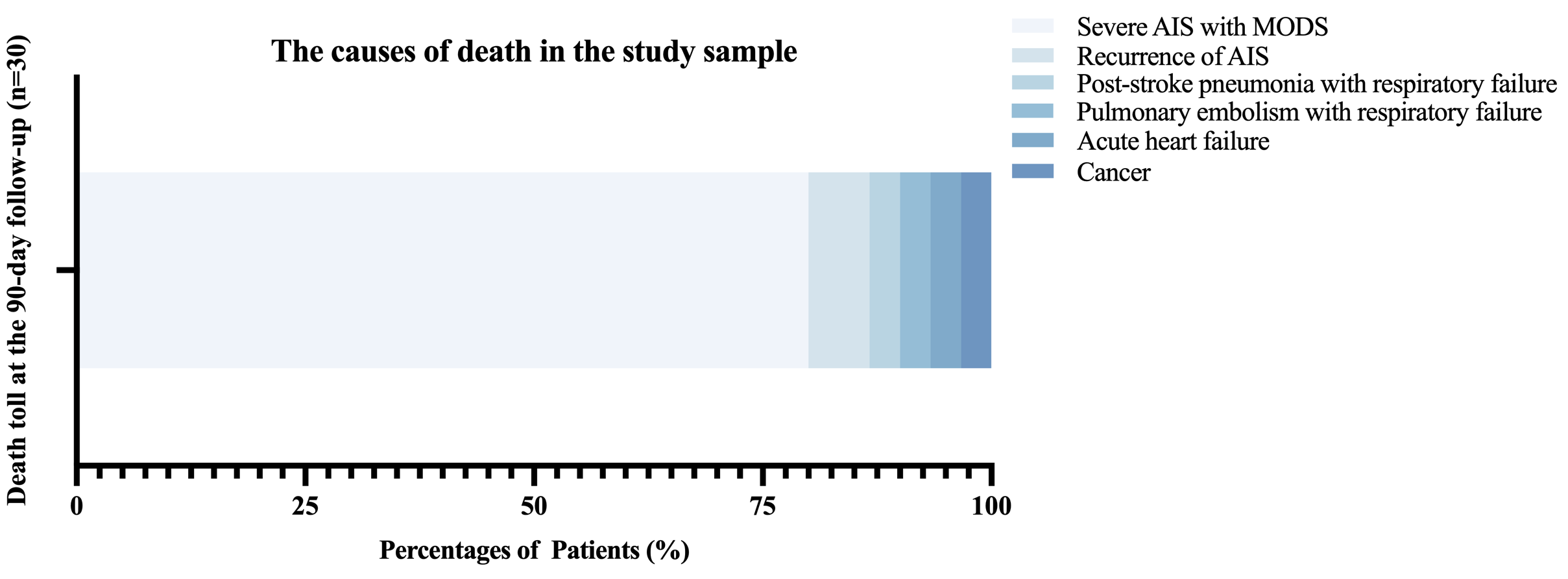

Supplement: Supplementary file 1 — Supplementary file1 (TIFF 178 KB) [file 415_2022_11515_MOESM1_ESM.tiff]
