# Supplementary material for: The optimum anticoagulation time after endovascular thrombectomy for atrial fibrillation-related large vessel occlusion stroke: a real-world study
Source: J Neurol. 2023 Jan 3;270(4):2084–95. doi: 10.1007/s00415-022-11515-y (PMC10025205; doi:10.1007/s00415-022-11515-y)
Supplement: Supplementary file 2 — Supplementary file2 (DOCX 13 KB) [file 415_2022_11515_MOESM2_ESM.docx]

**Figure Legend**

Supplemental Figure 1 The causes of death in the study sample.

A total of 30 patients who were included in this study died at 90-day follow-up. There were 24 (80%) patients who died from severe acute ischemic stroke (AIS) with multiple organ dysfunction syndromes (MODS), and 2 (1.5%) patients died from a recurrence of AIS. The causes of death included post-stroke pneumonia with respiratory failure, pulmonary embolism with respiratory failure, and acute heart failure cancer occurred in 1 (3.3%) patient in each.
